# Conceptual Models of Entrainment, Jet Lag, and Seasonality

**DOI:** 10.3389/fphys.2020.00334

**Published:** 2020-04-28

**Authors:** Isao T. Tokuda, Christoph Schmal, Bharath Ananthasubramaniam, Hanspeter Herzel

**Affiliations:** ^1^Department of Mechanical Engineering, Ritsumeikan University, Kyoto, Japan; ^2^Institute for Theoretical Biology, Humboldt Universität zu Berlin, Berlin, Germany; ^3^Institute for Theoretical Biology, Charité—Universitätsmedizin Berlin, Berlin, Germany

**Keywords:** circadian rhythms, amplitude-phase model, parameter optimization, jet lag, phase response curve, entrainment, seasonality

## Abstract

Understanding entrainment of circadian rhythms is a central goal of chronobiology. Many factors, such as period, amplitude, *Zeitgeber* strength, and daylength, govern entrainment ranges and phases of entrainment. We have tested whether simple amplitude-phase models can provide insight into the control of entrainment phases. Using global optimization, we derived conceptual models with just three free parameters (period, amplitude, and relaxation rate) that reproduce known phenotypic features of vertebrate clocks: phase response curves (PRCs) with relatively small phase shifts, fast re-entrainment after jet lag, and seasonal variability to track light onset or offset. Since optimization found multiple sets of model parameters, we could study this model ensemble to gain insight into the underlying design principles. We found complex associations between model parameters and entrainment features. *Arnold* onions of representative models visualize strong dependencies of entrainment on periods, relative *Zeitgeber* strength, and photoperiods. Our results support the use of oscillator theory as a framework for understanding the entrainment of circadian clocks.

## 1. Introduction

### 1.1. Entrainment and Oscillator Theory

The circadian clock can be regarded as a system of coupled oscillators. Examples include the neuronal network in the SCN (Hastings et al., 2018) and the “orchestra” of body clocks (Dibner et al., 2010). Furthermore, the intrinsic clock is entrained by *Zeitgebers*, such as light, temperature, and feeding. The concept of interacting oscillators (Van der Pol and Van der Mark, 1927; Kuramoto, 1984; Huygens, 1986; Strogatz, 2004) can contribute to the understanding of entrainment (Winfree, 1980). The theory of periodically driven self-sustained oscillators leads to the concept of “*Arnold* tongues” (Glass and Mackey, 1988; Pikovsky et al., 2003; Granada et al., 2009). *Arnold* tongues predict the ranges of periods and *Zeitgeber* strengths in which entrainment occurs (Abraham et al., 2010). The range of *Zeitgeber* periods over which entrainment occurs is called the “range of entrainment” (Aschoff and Pohl, 1978). If seasonal variations are also considered, the entrainment regions are termed “*Arnold* onions” (Schmal et al., 2015). Within these parameter regions, amplitudes and entrainment phases can vary drastically. Amplitude expansion due to external periodic driving is termed “resonance” (Duffing, 1918). Of central importance in chronobiology is the variability of the entrainment phase, since it allows the coordination of the intrinsic clock phase with the environment (Aschoff, 1960). Appropriate periods also provide evolutionary advantages (Ouyang et al., 1998; Dodd et al., 2005).

### 1.2. Phenomenological Amplitude-Phase Models

After the discovery of transcriptional feedback loops (Hardin et al., 1990), many mathematical models have focused on gene-regulatory networks (Forger and Peskin, 2003; Leloup and Goldbeter, 2003; Becker-Weimann et al., 2004). More recent models include many details of the transcriptional-translational feedback loops (Zhou et al., 2015; Bellman et al., 2018). However, most available data on phase response curves (PRCs) (Johnson, 1992), entrainment ranges (Aschoff and Pohl, 1978), and phases of entrainment (Rémi et al., 2010) are based on organismic data. Thus, it seems reasonable to study phenomenological models that are directly based on these empirical features. There is a long tradition of heuristic amplitude-phase models in chronobiology (Klotter, 1960; Wever, 1962; Pavlidis, 1973; Daan and Berde, 1978; Winfree, 1980; Kronauer et al., 1982).

Amplitude-phase models are quite generic and could be applied to any organism. We have adapted the entrainment features, discussed below in detail, to observed data of mammals (Daan and Aschoff, 1975; Reddy et al., 2002; Comas et al., 2006). Here, we have examined the capability of such heuristic amplitude-phase models to reproduce fundamental properties of circadian entrainment. To this end, we combine the traditional amplitude-phase modeling approach with recent oscillator theory and global optimization to identify minimal models that can reproduce essential features of mammalian clocks: PRCs with relatively small phase shifts (Honma et al., 2003), fast re-entrainment after jet lag (Yamazaki et al., 2000), and seasonal variability (Daan and Aschoff, 1975).

### 1.3. Entrainment Features and Model Constraints

Intrinsic periods of various organisms approximate in general the daylength of 24 h (Wyse et al., 2010). For example, *Neurospora* strains exhibit periods between 21 and 27 h (Lakin-Thomas et al., 1991; Merrow et al., 1999; Loros and Dunlap, 2001). Nocturnal rodents typically exhibit periods between 23 and 24 h in constant darkness (Pittendrigh and Daan, 1976a), whereas humans mostly exhibit periods slightly above 24 h (Wever, 1979; Czeisler et al., 1999). *Zeitgeber* signals, such as light, can accelerate or decelerate intrinsic rhythms leading to entrainment. PRCs quantify the amplitude and direction of phase shifts induced by *Zeitgeber* pulses (Wever, 1964; Johnson, 1992). In mammals, the strong coupling of SCN neurons constitutes a strong oscillator (Abraham et al., 2010; Granada et al., 2013), which has PRCs with relatively small phase shifts (Comas et al., 2006). Even bright light pulses of 6.7 h duration can shift the clock by just a few hours (Khalsa et al., 2003). These observations constitute the first constraint on our models. We assume that the maximal phase shifts are just 1 or 2 h.

Small phase shifts due to the *Zeitgeber* can lead to long transients of phase relaxation after jet lag (Kori et al., 2017). In many cases, a surprisingly fast recovery from jet lag is observed (Reddy et al., 2002; Vansteensel et al., 2003). These findings lead to our second model constraint. Along the lines of a previous optimization study (Locke et al., 2008), we request that our models reduce the jet lag-induced phase shift by 50 % within 2 days.

The third constraint refers to the well-known seasonal variability of circadian clocks (Pittendrigh and Daan, 1976b; Hazlerigg and Wagner, 2006; Rémi et al., 2010). It has been reported that phase markers can lock to dusk or dawn for varying daylengths. This implies that the associated phases change by 4 h, if we switch from 16:8 LD conditions to 8:16 LD conditions. Thus, we also optimize our models to exhibit such pronounced phase differences between 16:8 and 8:16 LD cycles.

After having introduced our model and the optimization procedure in the Methods section, we have tested whether or not simple amplitude-phase models can reproduce the three entrainment features discussed above.

## 2. Methods

### 2.1. Optimization of the Amplitude-Phase Model

As a model of an autonomous circadian clock, we consider the following amplitude-phase oscillator (Glass and Mackey, 1988; Abraham et al., 2010):

(1) $$\begin{array}{r} \frac{dr}{dt}=\lambda r\left(A-r\right), \end{array}$$

(2) $$\begin{array}{r} \frac{d\varphi }{dt}=\omega =\frac{2\pi }{\tau }. \end{array}$$

The system is described in polar coordinates by radius *r* and angle φ and has a limit cycle with amplitude *A* and angular frequency ω (or period τ). Any perturbation away from the limit cycle will relax back with a relaxation rate λ. This oscillator model can be represented in cartesian (*x, y*) coordinates as

(3) $$\begin{array}{r} \frac{dx}{dt}=-\lambda x\left(r-A\right)-\omega y+Z\left(t\right), \end{array}$$

(4) $$\begin{array}{r} \frac{dy}{dt}=-\lambda y\left(r-A\right)+\omega x, \end{array}$$

where $r=\sqrt{x^{2}+y^{2}}$. The oscillator receives a *Zeitgeber* signal

(5) $$\begin{array}{l} Z\left(t\right)=\{\begin{array}{ll} 1 & \text{if }t\;\mathrm{mod}\;T<\varkappa T\\ 0 & \text{otherwise}, \end{array} \end{array}$$

where *T* represents the period of the *Zeitgeber* signal, and ϰ determines the photoperiod (i.e., fraction of time during *T* hours when the lights are on). Amplitude-phase models provide the simplest mathematical framework to study limit cycle oscillations, which have been discussed in the context of circadian rhythms (Wever, 1962; Winfree, 1980; Kronauer et al., 1982).

The amplitude-phase model (1), (2) has three unknown parameters {*A*, ω, λ}. These parameters were optimized to satisfy the model constraints described in 1.3. The parameter optimization is based on the minimization of a cost function. The cost function takes a set of parameters as arguments, evaluates the model using those parameters, and then returns a “score” indicating the goodness of fit. Scores may only be positive, where a fit with score closer to zero represents a better fit. The cost function is given by

(6) $$\begin{array}{r} E\left(A,\omega ,\lambda \right)=\frac{{\left(T_{e}-48\text{h}\right)}^{2}}{{\left(24\text{h}\right)}^{2}}+\frac{{\left(\Delta \varphi_{max}-1\text{h}\right)}^{2}}{{\left(1\text{h}\right)}^{2}}+\frac{{\left(\Delta \psi -4\text{h}\right)}^{2}}{{\left(4\text{h}\right)}^{2}}, \end{array}$$

where *T*_*e*_, Δφ_*max*_, and Δψ represent half-time to re-entrainment, maximum phase-shift, and seasonal phase variability, respectively. The denominators can be regarded as tolerated ranges. If the values of *T*_*e*_, Δφ_*max*_, and Δψ deviate 24, 1, and 4 h from their target values, a score of three results. All parameter sets discussed in this paper had optimized scores below 0.1, i.e., the constrains are well-satisfied. Below we describe in detail how our quantities *T*_*e*_, Δφ_*max*_, and Δψ were calculated.

When the circadian oscillator is entrained to the *Zeitgeber* signal, their phase difference ψ = Ψ − φ (Ψ = 2π*t*/*T*: phase of the *Zeitgeber*) converges to a stable phase ψ_*e*_, which is called the “phase of entrainment.” The half-time to re-entrainment *T*_*e*_ denotes the amount of time required for the oscillator to recover from jet lag. As the *Zeitgeber* phase is advanced by ΔΨ, the phase difference becomes ψ = ψ_*e*_ + ΔΨ. *T*_*e*_ quantifies how long it takes until the advanced phase is reduced to less than half of the original shift due to jet lag (i.e., |ψ − ψ_*e*_| < 0.5ΔΨ). In our computation, this quantity was averaged over 24 different times during the day, at which 6h-advanced jet lag was applied. Next, the seasonal phase variability, which quantifies variability of the phase of entrainment over photoperiod from long day (16:8 LD) to short day (8:16 LD), is computed as Δψ = max_ϰ∈[1/3, 2/3]_ψ_*e*_ − min_ϰ∈[1/3, 2/3]_ψ_*e*_. Finally, the maximum phase-shift is given by Δφ_*max*_ = max_φ_|*PRC*(φ)|, where *PRC*(φ) represents PRC of the free-running oscillator, to which a 6 h light pulse is injected at its phase of φ.

To find optimal parameter values, the cost function was minimized by a particle swarm optimization algorithm (Eberhart and Kennedy, 1995; Trelea, 2003). Search ranges of the parameter values were set to *A*∈[0, 5], ω∈[2π/30 rad/h, 2π/18 rad/h], λ∈[0h^−1^, 0.5h^−1^]. Altogether 600 sets of parameter values were obtained. From the estimated parameters, the intrinsic period was obtained as τ = 2π/ω.

### 2.2. Simulations of Jet Lag, Phase Response, and Seasonality

**Figure 1:**
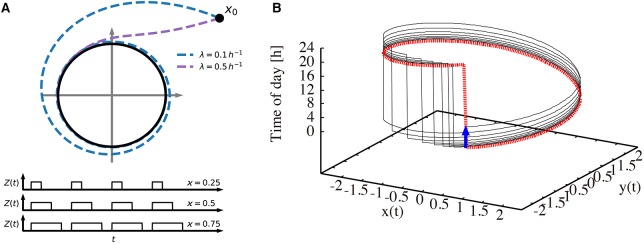
Visualization of the amplitude-phase model. **(A)** Schematic illustration of the amplitude-phase oscillator model (top) *Zeitgeber* signals (bottom) of different photoperiods ϰ. Starting from the initial condition *x*_0_, a small relaxation rate (λ = 0.1 h^−1^) gives rise to a long transient until its convergence back to the limit cycle, while transients for large relaxation rates (λ = 0.5 h^−1^) are short. **(B)** The re-entrainment process of the oscillator after its phase is shifted by a 6 h-advanced jet lag (thin black line). The thick dotted red line represents the trajectory that the system converges to. The thick blue arrow indicates the 6 h jet lag.

Figure 1 illustrates our modeling approach. In Figure 1A, the amplitude-phase equations (1) and (2) are visualized in the phase plane together with the driving *Zeitgeber* switching between 0 (dark) and 1 (light) for varying photoperiods. Two values of the amplitude relaxation rate λ illustrate how λ affects the decay of perturbations. Starting from an initial condition, a small relaxation rate gives rise to a long transient until its convergence to the limit cycle, while systems with large relaxation rates exhibit only a short transient. Figure 1B shows the oscillations in a 3-dimensional phase space. Two coordinates (*x* and *y*) span the phase plane of the endogenous oscillator. The vertical axis represents the phase of the *Zeitgeber*. The dotted red line marks the periodically driven limit cycle. The jump from 24 to 0 h reflects simply the periodic nature of our daily time.

**Figure 2:**
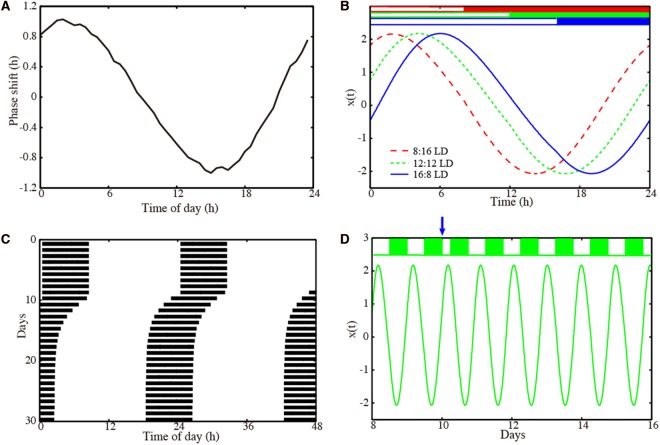
Properties of our amplitude-phase model for a representative optimized parameter set. **(A)** Phase response curve with respect to a 6 h light pulse. **(B)** Waveforms *x*(*t*) of the oscillator entrained to *Zeitgeber* signals with 8:16 LD (dashed red line), 12:12 LD (dotted green line), and 16:8 LD (solid blue line). **(C)** Actogram of the oscillator, to which a 6 h advancing jet lag was induced on day 10. **(D)** Time-trace *x*(*t*) of the oscillators, to which a 6 h advancing jet lag was induced on day 10. Model parameters: τ = 23.36 h, *A* = 2.063, λ = 0.386 h^−1^ with $\omega =\frac{2\pi }{\tau }$.

Interestingly, the relaxation after jet lag can be visualized as a transient convergence to the dotted red limit cycle via the black line after a 6 h phase change due to jet lag (blue arrow). Such a relaxation might be accompanied by amplitude changes (not apparent in the figure) and by steady phase shifts from day to day (note that the jump from 24 to 0 h is shifted day by day). After a few days, the dotted red line is approached, implying a vanishing jet lag. More conventional visualizations of the recovery from jet lag are given in Figure 2.

### 2.3. Global Sensitivity Analysis

To study the dependencies of the entrainment features on the model parameters, Sobol's global sensitivity analysis was carried out (Sobol, 1993, 2001; Morio, 2011). The global sensitivity indices computed by Monte Carlo simulations reveal how the input (model parameters) variability influences the variability in the output (entrainment features).

We have denoted an entrainment feature (i.e., re-entrainment time *T*_*e*_, maximum shift Δφ_*max*_, or seasonal variability Δψ) as *Y* = ϕ(*X*_1_, *X*_2_, *X*_3_) using a scalar function ϕ:ℝ^3^ → ℝ. The inputs {*X*_1_, *X*_2_, *X*_3_} stand for random variables that represent the model parameters {τ, *A*, λ}, respectively. The random variables are assumed to be uniformly distributed. The first-order sensitivity indices *S*_*i*_ for the input *X*_*i*_ (*i* = 1, 2, 3) are defined as *S*_*i*_ = *Var*(*E*(*Y*|*X*_*i*_))/*Var*(*Y*), where *Var* and *E* are variance and expectation, respectively. The second-order sensitivity indices *S*_*i*_ for the inputs *X*_*i*_ and *X*_*j*_ are defined as *S*_*ij*_ = {*Var*(*E*(*Y*|*X*_*i*_, *X*_*j*_)) − *Var*(*E*(*Y*|*X*_*i*_)) − *Var*(*E*(*Y*|*X*_*j*_))}/*Var*(*Y*). The total sensitivity indices *S*_*T*_*i*__ for the input *X*_*i*_ are finally given by $S_{T_{i}}=\sum_{k\#i}S_{k}$, where *#i* represents all the sets of indices that contain *i* (e.g., *S*_*T*_1__ = *S*_1_ + *S*_12_ + *S*_13_ + *S*_123_). These indices quantify the influence of the different inputs on the variance of *Y*. In our study, the Sobol indices were estimated with Monte-Carlo methods, where the number of randomly generated samples to estimate the indices was set to *N* = 10, 000.

## 3. Results and Discussions

### 3.1. Models Reproduce PRC With Small Phase Shifts, Short Jet Lag, and Seasonality

We performed 200 successful parameter optimizations leading to an ensemble of parameter sets. We analyzed, in this section, the parameters with a PRC having 1 h delay and advance. In Figures S1, S2, we also present parameter sets obtained with a modified optimization: in that case, we requested a PRC with a 2 h delay and advance. Figure 2 shows results for a representative model obtained via optimization. The PRC in Figure 2A is almost sinusoidal with maximal delays and advances of 1 h as requested by the optimization. Simulations with different photoperiods are shown in Figure 2B. It is evident that there are major phase shifts due to the varying photoperiods. The small-amplitude PRC implies that phase shifts by light are limited. Consequently, long transients after jet lag might be expected. Interestingly, Figure 2C visualizes a relatively fast recovery from jet lag. Figure 2D illustrates the re-entrainment after a jet lag applied on day 10. Note that no pronounced amplitude changes were observed.

It turns out that simple models with just three free parameters can successfully reproduce phenotypic features. In particular, fast recovery from jet lag for PRCs with quite small phase shifts is surprising. We next exploited the ensembles of parameter sets to understand the underlying principles.

### 3.2. Optimization Produced Highly Clustered Parameter Sets

In this section, we have focused on the 200 parameter sets with the ±1 h PRCs exemplified in Figure 2 (see Figure S1 for ±2 h PRCs).

The possible search ranges for our parameters were quite large (periods between 18 and 30 h, amplitudes between 0 and 5, and amplitude relaxation rates between 0 and 0.5h^−1^). The histograms from the optimized parameter sets demonstrate that the search leads to quite specific values: periods of around 23.3 ± 0.1 h, amplitudes of about 2.1 ± 0.04, and large relaxation rates of 0.25 ± 0.1h^−1^.

The optimized amplitude can be easily understood from the constraint on PRCs: for a given pulse strength the PRC shrinks monotonically with increasing amplitude (Pittendrigh, 1981; Vitaterna et al., 2006). A large amplitude of about 2.1 can be understood as a result of PRC having small phase shifts (±1 h). In contrast, our optimizations with a ±2 h PRC lead to smaller amplitudes around ±1.2 (see Figure S1b).

Amplitude relaxation rates range between 0.07 and 0.5 h^−1^. A value of 0.07 h^−1^ corresponds to a half-life of amplitude perturbations of about 10 h, while a value of 0.5 h^−1^ corresponds to a half-life of amplitude perturbations of about 1.4 h. Thus, all values in the histogram imply relatively fast amplitude relaxation. In Abraham et al. (2010), we termed limit cycles with fast amplitude relaxation “rigid oscillators.” Interestingly, Comas et al. (2007) found that two light pulses separated by 10 h shift phases almost independently. This finding confirms earlier studies of double pulses (Pittendrigh and Daan, 1976b). These observations are consistent with fast amplitude relaxation rates. Jet lag leads to a specific type of transient (compare Figure 1B). Thus, it seems reasonable that fast amplitude relaxation helps to achieve short transients after a jet lag.

The most surprising result of our optimization is the narrow range of intrinsic periods. We have argued that specific periods allow appropriate seasonal flexibility (compare Figure 4). In short, at specific parts of *Arnold* onions (i.e., the entrainment regions in the ϰ–*T* parameter plane), the required 4 h phase differences were found to give a reasonable phase shifts between 16:8 LD and 8:16 LD. We emphasize that specific periods (23.36 h in Figure 2, 24.64 h in Figure S2, 23.48 h in Figure S6) were not fitted to specific organisms.

**Figure 3:**
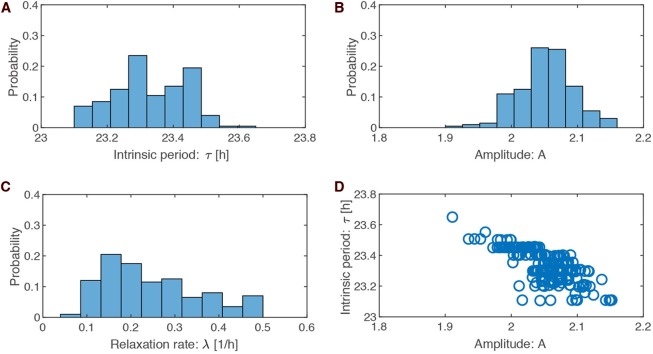
Parameter values in the optimized ensemble. **(A–C)** Distributions of the optimized parameter values for intrinsic period τ (= 2π/ω), oscillation amplitude *A*, and relaxation rate λ, respectively. **(D)** Scatter plots of amplitude *A* against intrinsic period τ drawn for the 200 sets of optimized parameters.

Figure 3D illustrates that the optimized parameter values are not independent. For instance, shorter periods are associated with larger amplitude. A possible explanation is that short periods imply larger effective pulse strength (a 6 h pulse is a larger part of a 23 h than a 24 h period) leading to larger amplitude in order to maintain the requested PRC amplitude.

In order to evaluate the robustness of our optimization approach, we generated also 200 parameter sets with PRCs with about a 2 h advance and delay. In these cases, we found intrinsic periods of 24.6 ± 0.1 h, amplitudes of 1.18 ± 0.08, and relaxation rates of 0.4 ± 0.1h^−1^. The relaxation rates and amplitude-period correlations were similar to the results with PRCs of about 1 h advance and delay (compare Figure 3 and Figure S1).

It should be noted that the PRC and the intrinsic period are in a trade-off relationship (Figures S3l, S4l, S5l). For a quick recovery from advanced jet lag, a short period (<24 h) is advantageous, since the jet lag-induced phase shift is reduced everyday by the period difference to 24 h. A long period (>24 h), on the other hand, requires a stronger *Zeitgeber* forcing and thus PRC with larger phase shifts, since the phase shift is otherwise increased by the period difference to 24 h. For this reason, ±1 h PRCs produced short periods, while ±2 h PRCs leaded to longer periods.

### 3.3. Complex Association Between Model Parameter and Entrainment Features

Our global optimization provided 200 sets of parameters {τ, *A*, λ} that reproduced the entrainment features of PRC with small phase shifts, fast recovery from jet lag, and high seasonal variability. Figures S3, S4 summarize the results for ±1 h PRCs and ±2 h PRCs, respectively. The upper 3 graphs show associations between the model parameters, illustrating that the parameters are not independent. The middle 9 graphs represent associations between the model parameters and the entrainment features (re-entrainment time *T*_*e*_, maximum shift Δφ_*max*_, seasonal variability Δψ), while the lower 3 graphs display associations between the entrainment features. The resulting patterns are quite complex and depend on specific constraints.

Since the recovery time from jet lag varies strongly even for mice from the same strain (Evans et al., 2015), we also optimized models for 3 day re-entrainment time instead of 2 day. The resulting scatter plots in Figure S5 reveal interesting changes: the intrinsic periods range from 23.4 h to 24.3 h and there exists a parameter set with a rather low relaxation time of 75 h. Thus, a relaxed jet lag constraint allows other periods and slow amplitude relaxation. Details regarding the parameter set with slow relaxation are provided in Figure S6. The recovery from jet lag is now accompanied by a small amplitude change as found in Goodwin models (Ananthasubramaniam et al., 2020) and the *Arnold* onion is less tilted as predicted (Schmal et al., 2015).

In order to quantify the complex associations of the model parameters and the entrainment features, we performed Sobol's global sensitivity analysis (Sobol, 1993, 2001; Morio, 2011). The strongest correlation was found between amplitude (*A*) and PRC size (Δφ_*max*_), as expected (Table 1). Re-entrainment time (*T*_*e*_) and seasonal variability (Δψ) are influenced by all the model parameters (τ, *A*, λ). Second-order sensitivities reveal that the relaxation rate (λ) effects re-entrainment time and seasonal variability in synergy with amplitude changes.

**Table 1:** Sobol's global sensitivity analysis, showing dependence of the entrainment features (re-entrainment time: *T*_*e*_, maximum shift: Δφ_*max*_, seasonal variability: Δψ) on the model parameters (intrinsic period: τ, amplitude: *A*, relaxation rate: λ).

	**Parameters**	**Re-entrainment time (T_*e*_)**	**Seasonality (Δψ)**	**PRC size (Δφ_*max*_)**
Total	τ	0.627	0.535	0.022
Sensitivity	*A*	0.724	0.777	0.823
*S*_*T*_*i*__	λ	0.335	0.295	0.006
First-order	τ	0.228	0.088	0.145
Sensitivity	*A*	0.174	0.233	0.967
*S*_*i*_	λ	0.077	0.085	−0.012
Second-order	τ, *A*	0.262	0.383	−0.118
Sensitivity	τ, λ	−0.029	0.049	0.044
*S*_*ij*_	*A*, λ	0.122	0.146	0.023

### 3.4. Arnold Onions Provide Insights Into the Optimized Parameters

**Figure 4:**
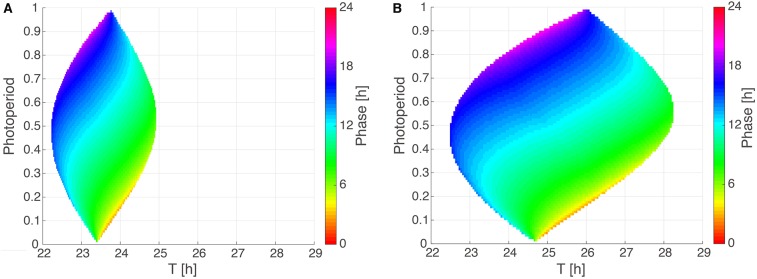
*Arnold* onions for the two PRC constraints. **(A,B)** 1:1 entrainment ranges in the ϰ-*T* parameter plane (*Arnold* onions). Entrainment phases were determined by numerical simulations and have been color-coded within the region of entrainment. Panel **(A)** depicts an *Arnold* onion for an optimized parameter set with a ±1 h PRC, a short-period τ = 23.36 h, *A* = 2.063, and λ = 0.386 h^−1^. Panel **(B)** shows an *Arnold* onion for an optimized parameter set with a ±2 h PRC, a long–period τ = 24.64 h, *A* = 1.144, λ = 0.50 h^−1^.

To systematically investigate the impact of photoperiod (ϰ) and Zeitgeber period (*T*) on entrainment properties, we analyze in Figure 4 two *Arnold* onions for representative parameter sets with a short period and a ±1 h PRC as well as a large period and a ±2 h PRC. Interestingly, the *Arnold* onions are tilted, i.e., the DD periods (constant darkness at photoperiod ϰ = 0) are smaller than the LL periods (constant light at photoperiod ϰ = 1) as predicted by *Aschoff's rule* for nocturnal animals (Aschoff, 1960). The largest entrainment range is found around a photoperiod of ϰ = 0.5 as predicted by Wever (1964). As expected, a larger PRC implies a wider range of entrainment (compare sizes of the *Arnold* onion in Figures 4A,B).

The phase of entrainment is color-coded in Figure 4. It is evident that the phases vary strongly with the *Zeitgeber* period *T*. There are theoretical predictions that phases change by about 12 h (Wever, 1964; Granada et al., 2013; Bordyugov et al., 2015) for different *Zeitgeber* periods. Interestingly, the variation of photoperiods in the vertical direction implies also very pronounced variations of the entrainment phase. Consequently, we could find many parameter sets with about 4 h phase shift between photoperiods of ϰ = 1/3 (8:16 LD) and of ϰ = 2/3 (16:8 LD).

## 4. Conclusions and Outlook

### 4.1. Optimized Models Reproduce Phenotypic Features

In most cases, the circadian clock of vertebrates is characterized by a relatively small type 1 PRC, by a narrow entrainment range, by fast recovery from jet lag, and by pronounced seasonal flexibility. We addressed whether or not these phenotypic features can be reproduced by simple amplitude-phase models with just three free parameters: period, amplitude, and relaxation time. To our surprise, we found many parameter sets via global optimization that reproduced the phenotypic features.

The availability of many parameter sets derived from random optimization allows extraction of essential properties of successful models. It turned out that the amplitude *A* is adjusted to reproduce PRCs with small phase shifts for a given *Zeitgeber* strength *Z*. According to limit cycle theory (Pavlidis, 1969; Peterson, 1980), the strength of a perturbation is governed by the ratio *Z*/*A* of *Zeitgeber* strength to amplitude. This implies that large limit cycles exhibit small phase shifts for a fixed *Zeitgeber* strength (Vitaterna et al., 2006).

In all suitable models, we found relatively fast amplitude relaxation rates with half-lives of perturbations below 10 h. This “rigidity” of limit cycles (Abraham et al., 2010) can support fast relaxation to the new phase after jet lag (compare Figure 1B). Double pulse experiments (Pittendrigh and Daan, 1976b; Comas et al., 2007) are consistent with fast relaxation rates.

In order to reproduce seasonality, we optimized our model under the constraint that 16:8 and 8:16 LD cycles have entrainment phases that are about 4 h apart from each other. This implies that the phase could follow dusk or dawn (Daan and Aschoff, 1975). In other words, we requested that the entrainment phase depends strongly on the photoperiod. As illustrated in Figure 4, such a strong dependency is indeed reproduced by our simple amplitude-phase models. Our optimization procedure selected specific periods that lead to a 4 h phase variation between photoperiods ϰ = 1/3 and ϰ = 2/3. Note that other periods can give large phase differences as well (compare the large vertical phase variations in Figure 4).

We have emphasized that our constraints were chosen to represent characteristic mammalian entrainment features: PRC with small phase shifts, relaxation from jet lag within a few days, and pronounced seasonal variability. Moreover, we based our constraints on light-pulse PRCs and considered only 6h-advanced jet lag. The observed increase of period with light intensity (compare Figure 4) resembles *Aschoff's rule* for nocturnal mammals.

Consequently, our results do not apply to clocks with type 0 PRCs and immediate phase resetting (Shaw and Brody, 2000; Devlin and Kay, 2001; Buhr et al., 2010). Furthermore, differences between phase advances and delays were not addressed. Other studies (Locke et al., 2005; Lu et al., 2016; Ananthasubramaniam et al., 2020) show that oscillator theory can also help to understand differences between advances and delays.

### 4.2. Relevance of Phenomenological Amplitude-Phase Models

Simplistic models as studied in this paper are quite generally applicable (Ananthasubramaniam et al., 2020). In principle, they could be used to describe single cells, tissue clocks, and organismic data. For single cells, damped stochastic oscillators can represent the observations also surprisingly well (Westermark et al., 2009). Such models have vanishingly small amplitudes and smaller relaxation rates, and they are driven by stochastic terms. Otherwise their complexity is comparable to our models discussed above.

The Poincaré model studied in this paper is particularly simple, since it has just three free parameters. Similar results on PRCs and entrainment have been described by other amplitude-phase models (Kronauer et al., 1982; Klerman et al., 1996; Flôres and Oda, 2020). The simplicity of our models implies that extensions are needed for fitting specific organisms and *Zeitgeber* profiles.

Complex models with multiple gene-regulatory feedback loops (Mirsky et al., 2009; Pokhilko et al., 2010; Relógio et al., 2011; Woller et al., 2016) could be reduced to amplitude-phase models simply by extracting periods, amplitudes, and relaxation rates from simulations. However, in such cases, the amplitudes are not uniquely defined since there are many dynamic variables.

### 4.3. How Can Circadian Amplitudes Be Defined?

This difficulty in defining amplitudes points to a general problem in chronobiology. Most studies focus on periods and entrainment phases. Limit cycle theory emphasizes that amplitudes are essential for understanding PRCs and entrainment. It is, however, not evident which amplitudes properly represent the limit cycle oscillator. Some studies consider gene expression levels (Lakin-Thomas et al., 1991; Wang et al., 2019) or reporter amplitudes (Leise et al., 2012), and other studies quantify activity rhythms (Bode et al., 2011; Erzberger et al., 2013). Since the ratio of *Zeitgeber* strength to amplitude *Z*/*A* governs PRCs and entrainment phases, we suggest that amplitudes could be quantified indirectly: the stronger the response to physiological perturbations, the smaller the amplitude. This approach leads to the concept of strong and weak oscillators (Abraham et al., 2010; Granada et al., 2013). Strong oscillators are robust and exhibit small phase shifts and narrow entrainment ranges but large phase variability (Granada et al., 2013). In this sense, wild-type vertebrate clocks represent strong oscillators in contrast to single cell organisms or plants. Indeed, the review of Aschoff and Pohl (1978) demonstrates impressively these properties.

Interestingly, a reduction in relative amplitudes (i.e., amplitudes as a fraction of the mesor) can reduce jet lag drastically, since resetting signals are much more efficient (An et al., 2013; Jagannath et al., 2013; Yamazaki et al., 2013).

### 4.4. Arnold Onions Quantify Entrainment

As shown in Figure 4, *Arnold* onions represent entrainment ranges and phases of entrainment in a compact manner. Astonishingly, even quite basic models lead to really complex variations of entrainment phases. As expected, the period mismatch *T*-τ has a rather strong effect on the entrainment phase. This reflects the well-known feature that short intrinsic periods τ have earlier entrainment phases (“chronotypes”) (Pittendrigh and Daan, 1976b; Merrow et al., 1999; Duffy et al., 2001). These associations are reflected in the horizontal phase variations in the *Arnold* onion. Interestingly, the vertical phase variability is also quite large. This observation demonstrates that also the effective *Zeitgeber* strength *Z*/*A* and the photoperiod affect the phase of entrainment strongly. Consequently, the expected correlations between periods and entrainment phase could be masked by varying amplitudes, *Zeitgeber* strength, and photoperiods. In other words, chronotypes are governed by periods only if relative *Zeitgeber* strength and photoperiod are kept constant.

The complexity of entrainment phase regulation indicates that generic properties of coupled oscillators can provide useful insights in chronobiology. In particular, basic amplitude-phase models can help understand the control of jet lag and seasonality.

## Data Availability Statement

All datasets generated for this study are included in the article/Supplementary Material.

## Author Contributions

HH designed the study. IT performed the model simulations. IT, BA, CS, and HH discussed the results. HH wrote the main text. IT, BA, and CS revised the text.

## Conflict of Interest

The authors declare that the research was conducted in the absence of any commercial or financial relationships that could be construed as a potential conflict of interest.

## Supplementary Material

Figure S1 Results of parameter optimization based on cost function $E\left(A,\omega ,\lambda \right)=\frac{{\left(T_{e}-48\text{h}\right)}^{2}}{{\left(24\text{h}\right)}^{2}}+\frac{{\left(\Delta \varphi_{max}-2\text{h}\right)}^{2}}{{\left(1\text{h}\right)}^{2}}+\frac{{\left(\Delta \psi -4\text{h}\right)}^{2}}{{\left(4\text{h}\right)}^{2}}$, where ±2 h PRC was requested. **(a–c)** Distributions of the 200 optimized parameter sets for τ (24.6±0.1 h), *A* (1.18±0.08), λ (0.4±0.1 h^−1^), respectively. **(d)** Scatter plots of amplitude *A* against intrinsic period τ drawn for 200 sets of optimized parameters.

Figure S2 Simulation of the amplitude-phase oscillator model using one of the 200 parameter sets optimized for a ±2 h PRC. **(a)** Phase response curve with respect to a 6 h light pulse. **(b)** Waveforms *x*(*t*) of the oscillator entrained to *Zeitgeber* signals with 8:16 LD (dashed red line), 12:12 LD (dotted green line), and 16:8 LD (solid blue line). **(c)** Actogram drawn for the oscillator, to which a 6 h advancing jet lag was induced on day 10. **(d)** Time-trace *x*(*t*) of the oscillators, to which a 6h advancing jet lag was induced on the 10th day. Parameter values: τ = 24.64 h, *A* = 1.144, λ = 0.50 h^−1^.

Figure S3 Scatter plots for 200 data sets optimized for ±1 h PRC with cost function $E\left(A,\omega ,\lambda \right)=\frac{{\left(T_{e}-48\text{h}\right)}^{2}}{{\left(24\text{h}\right)}^{2}}+\frac{{\left(\Delta \varphi_{max}-1\text{h}\right)}^{2}}{{\left(1\text{h}\right)}^{2}}+\frac{{\left(\Delta \psi -4\text{h}\right)}^{2}}{{\left(4\text{h}\right)}^{2}}.$ **(a)** Amplitude *A* vs. relaxation rate λ. **(b)** Amplitude *A* vs. intrinsic period τ. **(c)** Relaxation rate λ vs. intrinsic period τ. **(d)** Amplitude *A* vs. re-entrainment time *T*_*e*_. **(e)** Amplitude *A* vs. phase variability Δψ. **(f)** Amplitude *A* vs. maximum PRC Δφ_*max*_. **(g)** Relaxation rate λ vs. re-entrainment time *T*_*e*_. **(h)** Relaxation rate λ vs. phase variability Δψ. **(i)** Relaxation rate λ vs. maximum PRC Δφ_*max*_. **(j)** Intrinsic period τ vs. re-entrainment time *T*_*e*_. **(k)** Intrinsic period τ vs. phase variability Δψ. **(l)** Intrinsic period τ vs. maximum PRC Δφ_*max*_. **(m)** Re-entrainment time *T*_*e*_ vs. phase variability Δψ. **(n)** Re-entrainment time *T*_*e*_ vs. maximum PRC Δφ_*max*_. **(o)** Phase variability Δψ vs. maximum PRC Δφ_*max*_.

Figure S4 Scatter plots for 200 data sets optimized for ±2 h PRC with cost function $E\left(A,\omega ,\lambda \right)=\frac{{\left(T_{e}-48\text{h}\right)}^{2}}{{\left(24\text{h}\right)}^{2}}+\frac{{\left(\Delta \varphi_{max}-2\text{h}\right)}^{2}}{{\left(1\text{h}\right)}^{2}}+\frac{{\left(\Delta \psi -4\text{h}\right)}^{2}}{{\left(4\text{h}\right)}^{2}}.$ **(a)** Amplitude *A* vs. relaxation rate λ. **(b)** Amplitude *A* vs. intrinsic period τ. **(c)** Relaxation rate λ vs. intrinsic period τ. **(d)** Amplitude *A* vs. re-entrainment time *T*_*e*_. **(e)** Amplitude *A* vs. phase variability Δψ. **(f)** Amplitude *A* vs. maximum PRC Δφ_*max*_. **(g)** Relaxation rate λ vs. re-entrainment time *T*_*e*_. **(h)** Relaxation rate λ vs. phase variability Δψ. **(i)** Relaxation rate λ vs. maximum PRC Δφ_*max*_. **(j)** Intrinsic period τ vs. re-entrainment time *T*_*e*_. **(k)** Intrinsic period τ vs. phase variability Δψ. **(l)** Intrinsic period τ vs. maximum PRC Δφ_*max*_. **(m)** Re-entrainment time *T*_*e*_ vs. phase variability Δψ. **(n)** Re-entrainment time *T*_*e*_ vs. maximum PRC Δφ_*max*_. **(o)** Phase variability Δψ vs. maximum PRC Δφ_*max*_.

Figure S5 Scatter plots for 200 data sets optimized for ±1 h PRC and 3 days re-entrainment time with cost function $E\left(A,\omega ,\lambda \right)=\frac{{\left(T_{e}-72\text{h}\right)}^{2}}{{\left(24\text{h}\right)}^{2}}+\frac{{\left(\Delta \varphi_{max}-1\text{h}\right)}^{2}}{{\left(1\text{h}\right)}^{2}}+\frac{{\left(\Delta \psi -4\text{h}\right)}^{2}}{{\left(4\text{h}\right)}^{2}}.$ **(a)** Amplitude *A* vs. relaxation rate λ. **(b)** Amplitude *A* vs. intrinsic period τ. **(c)** Relaxation rate λ vs. intrinsic period τ. **(d)** Amplitude *A* vs. re-entrainment time *T*_*e*_. **(e)** Amplitude *A* vs. phase variability Δψ. **(f)** Amplitude *A* vs. maximum PRC Δφ_*max*_. **(g)** Relaxation rate λ vs. re-entrainment time *T*_*e*_. **(h)** Relaxation rate λ vs. phase variability Δψ. **(i)** Relaxation rate λ vs. maximum PRC Δφ_*max*_. **(j)** Intrinsic period τ vs. re-entrainment time *T*_*e*_. **(k)** Intrinsic period τ vs. phase variability Δψ. **(l)** Intrinsic period τ vs. maximum PRC Δφ_*max*_. **(m)** Re-entrainment time *T*_*e*_ vs. phase variability Δψ. **(n)** Re-entrainment time *T*_*e*_ vs. maximum PRC Δφ_*max*_. **(o)** Phase variability Δψ vs. maximum PRC Δφ_*max*_.

Figure S6 Entrainment features of the amplitude-phase model for τ = 23.48 h, *A* = 2.458, λ = 0.0134 h^−1^ with ω = 0.268. **(a)** The re-entrainment process of the oscillator after its phase is shifted by a 6 h–advanced jet lag. The red line represents the trajectory that the system converges to. **(b)** Phase response curve with respect to a 6h light pulse. **(c)** Waveforms *x*(*t*) of the oscillator entrained to *Zeitgeber* signal with 8:16 LD (purple), 12:12 LD (green), and 16:8 LD (blue). **(d)** Actogram drawn for the oscillator, to which a 6 h-advanced jet lag was induced on day 10. **(e)** Time-trace *x*(*t*) of the oscillators, to which a 6 h-advanced jet lag was induced on day 10. **(f)** *Arnold* onion (1:1 entrainment ranges in the ϰ-*T* parameter plane).
