# Supplementary figures and images for: Conceptual Models of Entrainment, Jet Lag, and Seasonality
Source: Front Physiol. 2020 Apr 28;11:334. doi: 10.3389/fphys.2020.00334 (PMC7199094; doi:10.3389/fphys.2020.00334)

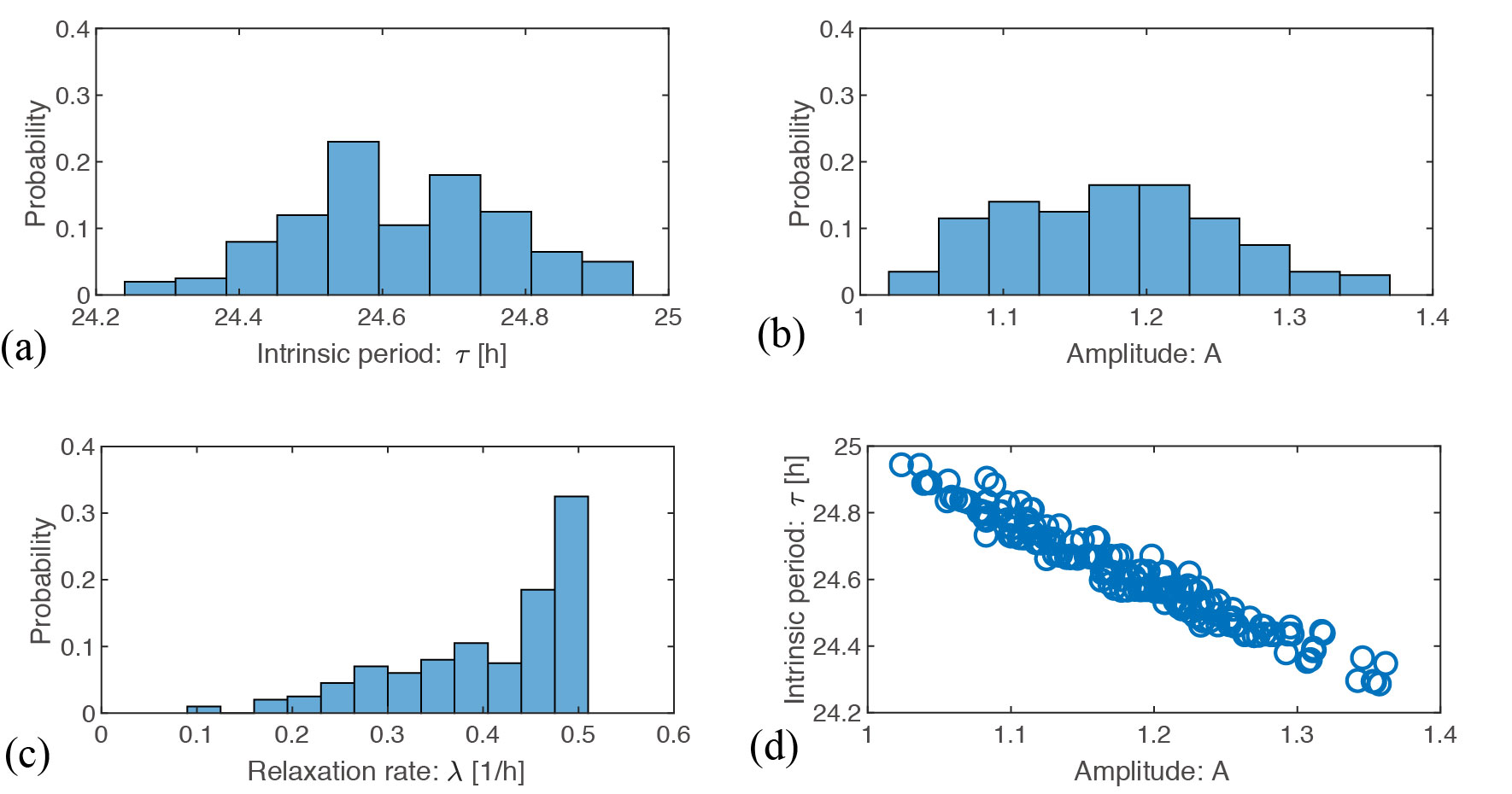

Supplement: Figure S1 — Results of parameter optimization based on cost function E(A,ω,λ)=(Te-48h)2(24h)2+(Δφmax-2h)2(1h)2+(Δψ-4h)2(4h)2, where ±2 h PRC was requested. (a–c) Distributions of the 200 optimized parameter sets for τ (24.6±0.1 h), A (1.18±0.08), λ (0.4±0.1 h−1), respectively. (d) Scatter plots of amplitude A against intrinsic period τ drawn for 200 sets of optimized parameters. [file Image_1.JPEG]

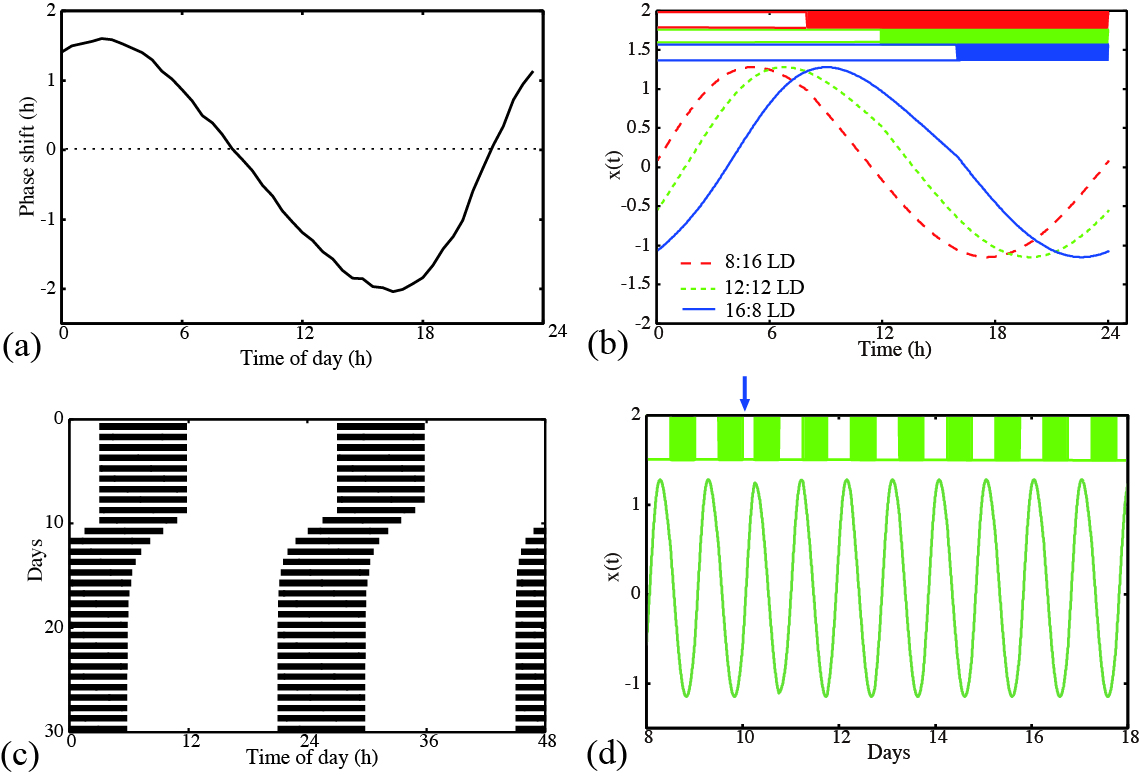

Supplement: Figure S2 — Simulation of the amplitude-phase oscillator model using one of the 200 parameter sets optimized for a ±2 h PRC. (a) Phase response curve with respect to a 6 h light pulse. (b) Waveforms x(t) of the oscillator entrained to Zeitgeber signals with 8:16 LD (dashed red line), 12:12 LD (dotted green line), and 16:8 LD (solid blue line). (c) Actogram drawn for the oscillator, to which a 6 h advancing jet lag was induced on day 10. (d) Time-trace x(t) of the oscillators, to which a 6h advancing jet lag was induced on the 10th day. Parameter values: τ = 24.64 h, A = 1.144, λ = 0.50 h−1. [file Image_2.JPEG]

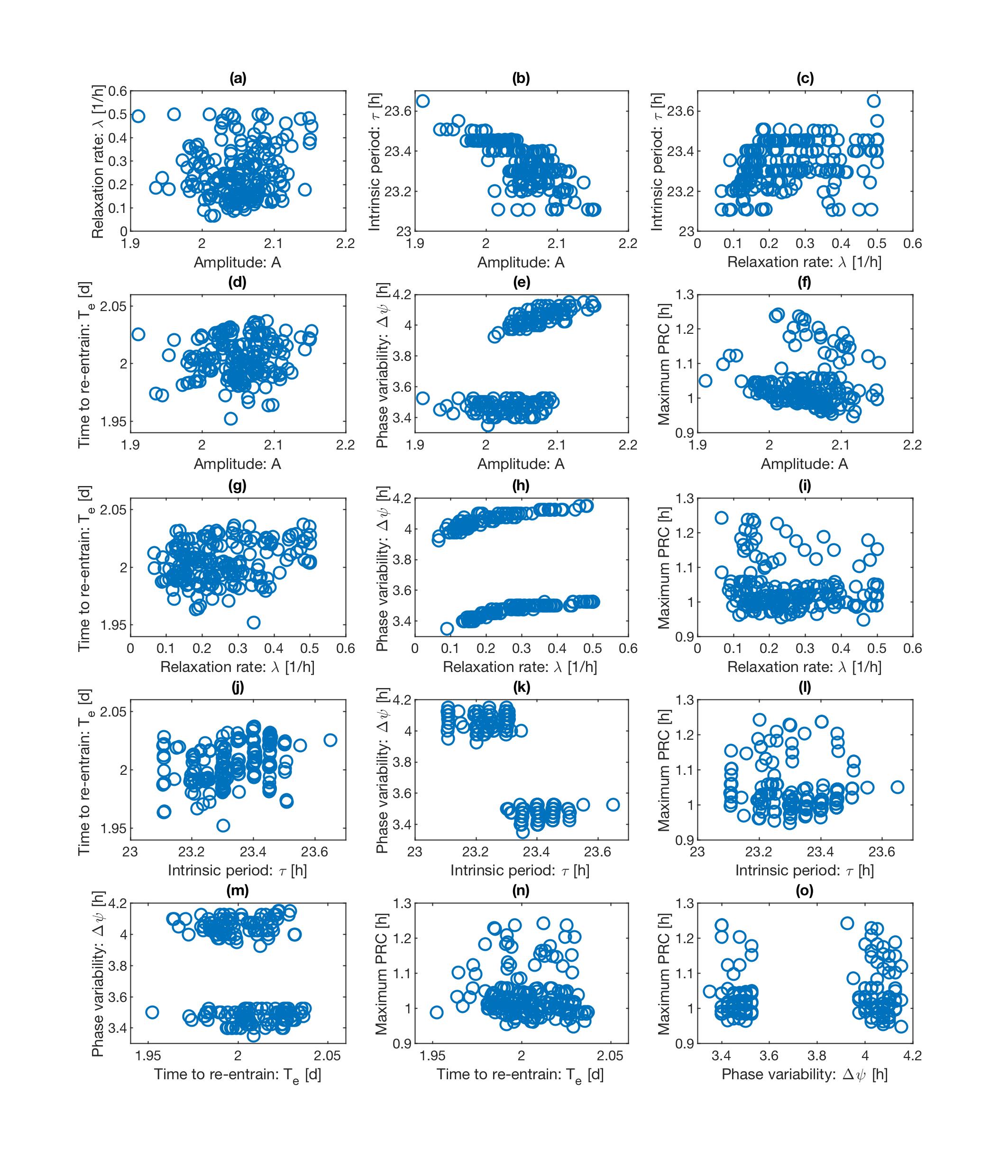

Supplement: Figure S3 — Scatter plots for 200 data sets optimized for ±1 h PRC with cost function E(A,ω,λ)=(Te-48h)2(24h)2+(Δφmax-1h)2(1h)2+(Δψ-4h)2(4h)2. (a) Amplitude A vs. relaxation rate λ. (b) Amplitude A vs. intrinsic period τ. (c) Relaxation rate λ vs. intrinsic period τ. (d) Amplitude A vs. re-entrainment time Te. (e) Amplitude A vs. phase variability Δψ. (f) Amplitude A vs. maximum PRC Δφmax. (g) Relaxation rate λ vs. re-entrainment time Te. (h) Relaxation rate λ vs. phase variability Δψ. (i) Relaxation rate λ vs. maximum PRC Δφmax. (j) Intrinsic period τ vs. re-entrainment time Te. (k) Intrinsic period τ vs. phase variability Δψ. (l) Intrinsic period τ vs. maximum PRC Δφmax. (m) Re-entrainment time Te vs. phase variability Δψ. (n) Re-entrainment time Te vs. maximum PRC Δφmax. (o) Phase variability Δψ vs. maximum PRC Δφmax. [file Image_3.JPEG]

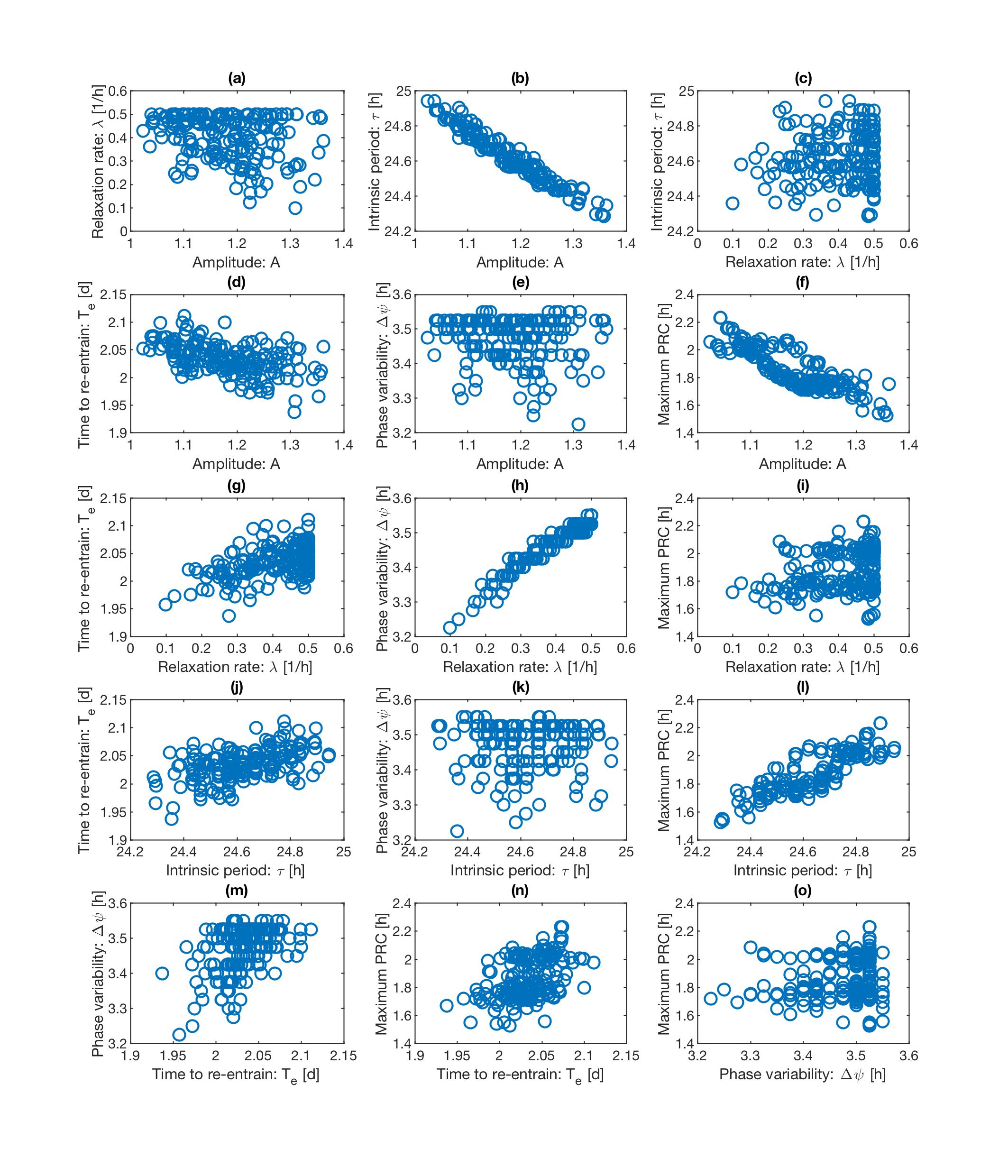

Supplement: Figure S4 — Scatter plots for 200 data sets optimized for ±2 h PRC with cost function E(A,ω,λ)=(Te-48h)2(24h)2+(Δφmax-2h)2(1h)2+(Δψ-4h)2(4h)2. (a) Amplitude A vs. relaxation rate λ. (b) Amplitude A vs. intrinsic period τ. (c) Relaxation rate λ vs. intrinsic period τ. (d) Amplitude A vs. re-entrainment time Te. (e) Amplitude A vs. phase variability Δψ. (f) Amplitude A vs. maximum PRC Δφmax. (g) Relaxation rate λ vs. re-entrainment time Te. (h) Relaxation rate λ vs. phase variability Δψ. (i) Relaxation rate λ vs. maximum PRC Δφmax. (j) Intrinsic period τ vs. re-entrainment time Te. (k) Intrinsic period τ vs. phase variability Δψ. (l) Intrinsic period τ vs. maximum PRC Δφmax. (m) Re-entrainment time Te vs. phase variability Δψ. (n) Re-entrainment time Te vs. maximum PRC Δφmax. (o) Phase variability Δψ vs. maximum PRC Δφmax. [file Image_4.JPEG]

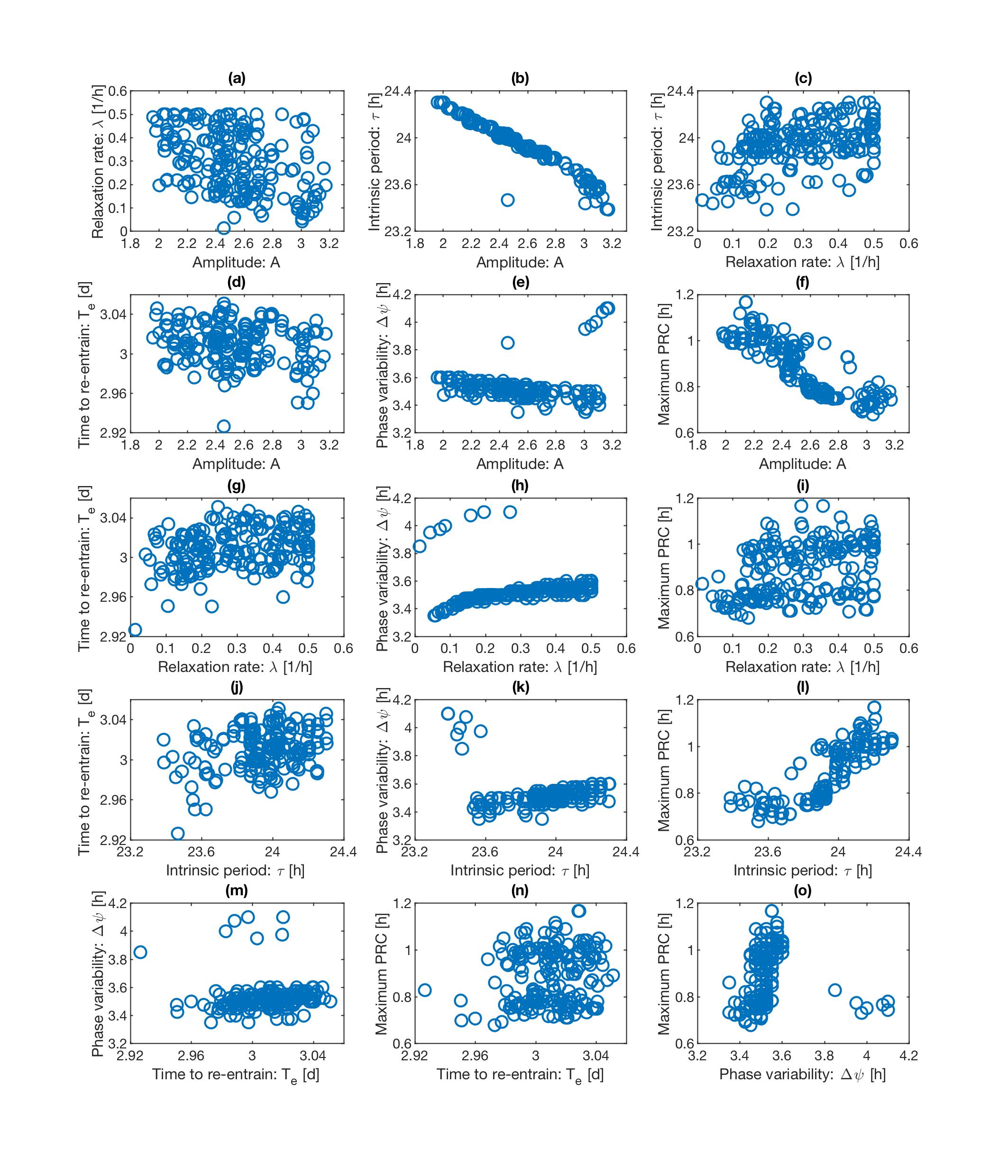

Supplement: Figure S5 — Scatter plots for 200 data sets optimized for ±1 h PRC and 3 days re-entrainment time with cost function E(A,ω,λ)=(Te-72h)2(24h)2+(Δφmax-1h)2(1h)2+(Δψ-4h)2(4h)2. (a) Amplitude A vs. relaxation rate λ. (b) Amplitude A vs. intrinsic period τ. (c) Relaxation rate λ vs. intrinsic period τ. (d) Amplitude A vs. re-entrainment time Te. (e) Amplitude A vs. phase variability Δψ. (f) Amplitude A vs. maximum PRC Δφmax. (g) Relaxation rate λ vs. re-entrainment time Te. (h) Relaxation rate λ vs. phase variability Δψ. (i) Relaxation rate λ vs. maximum PRC Δφmax. (j) Intrinsic period τ vs. re-entrainment time Te. (k) Intrinsic period τ vs. phase variability Δψ. (l) Intrinsic period τ vs. maximum PRC Δφmax. (m) Re-entrainment time Te vs. phase variability Δψ. (n) Re-entrainment time Te vs. maximum PRC Δφmax. (o) Phase variability Δψ vs. maximum PRC Δφmax. [file Image_5.JPEG]

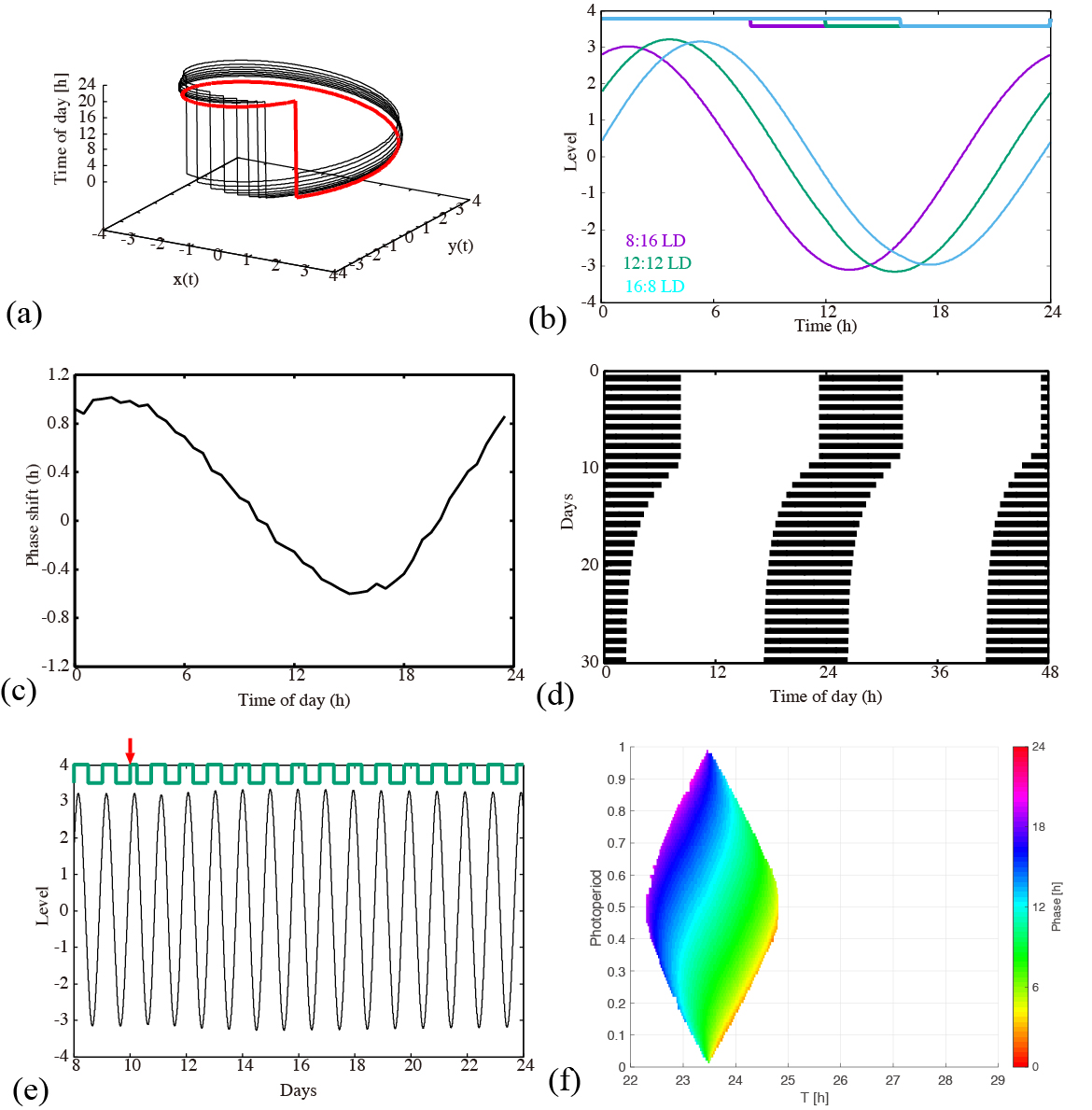

Supplement: Figure S6 — Entrainment features of the amplitude-phase model for τ = 23.48 h, A = 2.458, λ = 0.0134 h−1 with ω = 0.268. (a) The re-entrainment process of the oscillator after its phase is shifted by a 6 h–advanced jet lag. The red line represents the trajectory that the system converges to. (b) Phase response curve with respect to a 6h light pulse. (c) Waveforms x(t) of the oscillator entrained to Zeitgeber signal with 8:16 LD (purple), 12:12 LD (green), and 16:8 LD (blue). (d) Actogram drawn for the oscillator, to which a 6 h-advanced jet lag was induced on day 10. (e) Time-trace x(t) of the oscillators, to which a 6 h-advanced jet lag was induced on day 10. (f) Arnold onion (1:1 entrainment ranges in the ϰ-T parameter plane). [file Image_6.JPEG]
